# Supplementary material for: The Effect of FG-Nup Phosphorylation on NPC Selectivity: A One-Bead-Per-Amino-Acid Molecular Dynamics Study
Source: Int J Mol Sci. 2019 Jan 30;20(3):596. doi: 10.3390/ijms20030596 (PMC6387328; doi:10.3390/ijms20030596)
Supplement: Supplementary file 1 [file ijms-20-00596-s001.docx]

Supplementary Materials

**Table s1.** Source data for the $R_{S}$ of different FG-nup segmentsin the native state from experiments [36] and from simulations [33] and for the phosphorylated state (Phos_Max and Phos_N scenarios) from simulations. The errors *σ* reported here for the simulations represent the standard deviation of $R_{S}$ over the entire simulation time (Note that the FG-Nup simulations performed in [33] have been repeated here to quantify the error and thus some differences appear. These differences, however, are very small and fall well within the error bar). The symbol *** in the 3^rd^ and 4^th^ column stands for experiments and the other columns show data from the simulations.

| FG-Nup | *N* | $R_{S}^{native*}$ | $\sigma^{native*}$ | $R_{S}^{\mathrm{native}}$ | $\sigma^{\mathrm{native}}$ | $R_{S}^{Phos\_Max}$ | $\sigma^{Phos\_Max}$ | $R_{S}^{Phos\_N}$ | $\sigma^{Phos\_N}$ |
| --- | --- | --- | --- | --- | --- | --- | --- | --- | --- |
| Nsp1n_lc | 172 | 27.10 | 0.00 | 31.37 | 3.12 | 47.44 | 2.21 | 39.7 | 2.63 |
| Nup116m_lc | 551 | 46.50 | 0.00 | 44.13 | 3.54 | 83.55 | 5.16 | 50.67 | 4.11 |
| Nup100n_lc | 625 | 48.70 | 0.40 | 47.77 | 4.17 | 101.17 | 5.91 | 76.04 | 5.93 |
| Nup49_lc | 215 | 26.90 | 0.00 | 32.45 | 3.08 | 49.80 | 2.62 | 37.02 | 3.23 |
| Nup42_lc | 212 | 28.40 | 0.50 | 28.59 | 2.41 | 52.37 | 2.60 | 41.85 | 3.24 |
| Nup57_lc | 255 | 31.90 | 1.00 | 33.70 | 3.33 | 56.73 | 3.03 | 41.14 | 3.57 |
| Nup145n_lc | 242 | 28.20 | 0.20 | 30.68 | 2.58 | 56.06 | 2.98 | 43.33 | 3.43 |
| Nup1c_lc | 279 | 32.40 | 0.40 | 34.93 | 3.23 | 61.17 | 3.44 | 48.68 | 3.81 |
| Nup159_hc | 441 | 55.40 | 0.20 | 60.40 | 4.96 | 87.04 | 4.98 | 76.36 | 4.89 |
| Nup60_hc | 151 | 31.30 | 0.20 | 34.36 | 3.07 | 41.85 | 2.21 | 37.08 | 2.55 |
| Nup1m_hc | 578 | 67.90 | 0.20 | 71.22 | 5.70 | 96.84 | 6.96 | 80.41 | 6.02 |
| Nup2_hc | 376 | 59.80 | 0.30 | 54.82 | 5.14 | 77.14 | 4.75 | 65.17 | 4.8 |
| `Nsp1m_hc | 431 | 65.30 | 0.10 | 65.40 | 5.44 | 79.07 | 4.73 | 71.28 | 5.07 |
| Nup145ns | 191 | 29.80 | 0.00 | 35.83 | 2.60 | 48.73 | 2.57 | 43.58 | 2.82 |
| Nup100s | 190 | 36.60 | 0.30 | 40.83 | 3.33 | 49.30 | 2.51 | 43.31 | 2.63 |
| Nup116s | 196 | 39.10 | 0.20 | 42.22 | 3.35 | 46.55 | 2.84 | 42.48 | 3.3 |

**Table s2.** Amino acid composition of the studied FG-Nup segments. *N* denotes the total number of residues in the FG-Nup segments [36]. The suffix *lc* denotes low charge, *hc* high charge and *s* refers to the stalk region of the Nup, *m*, *n* and *c* represents the middle, N-terminal and C-terminal portions of the FG-segments [36]. The percentage of phosphorylatable residues for the Phos_N and Phos_Max cases are denoted by *n_*_Phos_N_ and *n_*_Phos_Max_, respectively. For Phos_N we use the NetPhosYeast 1.0 server [37] based on the default threshold score of 0.5. The fraction of positively-charged residues is represented by *p* and PRO represents the percentage of amino acid Proline. The net hydrophobicity for the FG-Nups was evaluated by adding the hydrophobicity values $\varepsilon_{1BPA, i}$ (from [33], see Methods section) of all residues, and we used this to calculate the phosphorylation-induced net decrease in hydrophobicity normalized with the hydrophobicity in the native state, shown in the ∆*H*_Phos_N_ and ∆*H*_Phos_Max_ columns for the Phos_N and Phos_Max cases, respectively.

| FG-Nup | *N* | *n_*_Phos_N_ | *n_*_Phos_Max_ | *p* | PRO | | ∆*H*_Phos_N_ | ∆*H*_Phos_Max_ |
| --- | --- | --- | --- | --- | --- | --- | --- | --- |
| Nsp1n_lc | 172 | 15% | 33% | 2% | | 4% | 10% | 20% |
| Nup116m_lc | 551 | 5% | 21% | 2% | | 3% | 3% | 13% |
| Nup100n_lc | 625 | 11% | 27% | 2% | | 3% | 7% | 17% |
| Nup49_lc | 215 | 7% | 25% | 3% | | 4% | 5% | 15% |
| Nup42_lc | 212 | 12% | 30% | 3% | | 8% | 8% | 18% |
| Nup57_lc | 255 | 9% | 28% | 3% | | 5% | 6% | 17% |
| Nup145n_lc | 242 | 12% | 30% | 3% | | 6% | 8% | 18% |
| Nup1c_lc | 279 | 12% | 28% | 3% | | 6% | 8% | 17% |
| Nup159_hc | 441 | 17% | 31% | 7% | | 8% | 10% | 22% |
| Nup60_hc | 151 | 11% | 22% | 12% | | 7% | 8% | 16% |
| Nup1m_hc | 578 | 9% | 26% | 14% | | 8% | 7% | 19% |
| Nup2_hc | 376 | 13% | 28% | 13% | | 8% | 10% | 21% |
| Nsp1m_hc | 431 | 9% | 21% | 16% | | 8% | 8% | 17% |
| Nup145ns | 191 | 15% | 25% | 13% | | 7% | 12% | 18% |
| Nup100s | 190 | 15% | 28% | 13% | | 6% | 12% | 21% |
| Nup116s | 196 | 4% | 15% | 17% | | 3% | 4% | 12% |

**Table s3.** Normalized hydrophobicity $\varepsilon_{k, i}$ (with respect to the most hydrophobic AA) for S, H, T and Y from the five hydrophobicity-predictor programs and $\varepsilon_{1BPA,i}$ from the 1BPA model [33].

| **AA** | **KOWWIN** | **ClogP** | **ChemAxon** | **ALOGPS** | **miLogP** | **1BPA** |
| --- | --- | --- | --- | --- | --- | --- |
| S | 0.41 | 0.42 | 0.13 | 0.13 | 0.04 | 0.45 |
| H | 0.47 | 0.00 | 0.21 | 0.25 | 0.28 | 0.53 |
| T | 0.52 | 0.57 | 0.26 | 0.28 | 0.17 | 0.51 |
| Y | 0.86 | 0.70 | 0.88 | 0.52 | 0.77 | 0.82 |

.

**Table s4.** Weights $w_{k,i}$ assigned to S, H, T and Y for the five hydrophobicity-predictor programs. The residue-specific weight assignment is based on how close each hydrophobicity-predictor program estimates the hydrophobicity of the amino acids in their native state (i.e., $\varepsilon_{k, i}$ in Table s3) compared to the 1BPA model [33] (i.e., $\varepsilon_{1BPA,i}$ in Table s3).

| **AA** | **KOWWIN** | **ClogP** | **ChemAxon** | **ALOGPS** | **miLogP** |
| --- | --- | --- | --- | --- | --- |
| S | 0.35 | 0.63 | 0.01 | 0.01 | 0.00 |
| H | 0.87 | 0.01 | 0.03 | 0.04 | 0.05 |
| T | 0.97 | 0.03 | 0.00 | 0.00 | 0.00 |
| Y | 0.48 | 0.05 | 0.21 | 0.01 | 0.25 |

**Table s5.** Physical properties for the wild type and phosphorylated NPC. For the net hydrophobicity we added the hydrophobicity values of all residues inside the NPC.

| **Forcefield** | **+ve charge** | **-ve charge** | **Net Charge** | **Net hydrophobicity** | **Phosphorylated AA** |
| --- | --- | --- | --- | --- | --- |
| Wild type | 8072 | -7560 | 512 | 43373.7 | 0 |
| Phos_N | 8072 | -20424 | -12352 | 41010.1 | 6432 |
| Phos_Max | 8072 | -49544 | -41472 | 36310.6 | 20992 |

***Sensitivity analysis:***

There exist other databases for the extraction of phosphorylatable sites, such as the Fungi phosphorylation database (FPD) [38], which comprises high-confidence in vivo phosphosites identified by MS-based proteomics. FPD predicts a lower number of phosphorylatable residues compared to the Phos_N scenario for the individual FG-Nup segments (see table s6) and the NPC (see table s7). However, we cannot be sure that all the phosphorylation sites predicted by FPD (referred to as the Phos_FPD scenario) are indeed phosphorylated simultaneously inside the NPC, an issue that also exists for the Phos_N and Phos_Max scenarios. In fact, it is unlikely that they are. We addressed the effect of phosphorylation of the FPD phosphosites from the insights gained from the Phos_Max and Phos_N results, without performing additional simulations for isolated FG Nups and passive transport. Such extrapolations are less straight-forward for active transport and we therefore conducted separate MD simulations for the Phos_FPD scenario.

The Phos_Max scenario predicts 2 to 4 times more phosphorylation sites compared to the Phos_N scenario (see Table s2), so that the distributions of the phosphorylated sites are different in both cases. Despite the differences between Phos_Max and Phos_N, we notice that FG-Nup segments from each family (*lc*, *hc* and *s*) show very similar slopes in the plots for the normalized change in Stokes radius as a function of the fraction of phosphorylatable residues (see Figure 2a and b), indicating that the slopes are not sensitive to the distribution of phosphorylation sites. This leads to an accurate fit of the simulations by the theoretical relation in Eq. (1), depicted in Fig. 2d. In table s6 we show the fraction of phosphorylatable residues extracted for the Phos_FPD scenario (*n_*_Phos_FPD_) and the predicted Stokes radius ($R_{S}^{Phos\_FPD})$ using Eqn 1. As the Phos_FPD scenario predicts a lower number of phosphorylatable residues compared to the Phos_N scenario, the predicted Stokes radii for Phos_FPD (see table s6) have intermediate values between that predicted for the FG-Nup segments in their native state and the Phos_N state (table s1).

**Table s6.** The Stokes radius predicted (in Angstrom) using Eqn. 1 for isolated FG-Nup segments under the Phos_FPD condition ($R_{S}^{Phos\_FPD}$), where the phosphorylation sites are extracted from the Fungi phosphorylation database (FPD). *n_*_Phos_FPD_ denotes the total fraction of phosphorylatable residues for the Phos_FPD scenario. As the fraction of phosphorylation sites is lower than that of the Phos_N scenario (see table s2), the predicted Stokes radii of all FG-Nup segments are inbetween the values predicted for the wild type and the Phos_N scenario in table s1.

| FG-Nup | *n_*_Phos_FPD_ | $R_{S}^{Phos\_FPD}$ | |
| --- | --- | --- | --- |
| Nsp1n_lc | 2% | 32.42 |  |
| Nup116m_lc | 0% | 44.13 |  |
| Nup100n_lc | 0% | 48.45 |  |
| Nup49_lc | NA | - |  |
| Nup42_lc | 1% | 29.59 |  |
| Nup57_lc | 3% | 36.05 |  |
| Nup145n_lc | 1% | 31.35 |  |
| Nup1c_lc | 0% | 34.93 |  |
| Nup159_hc | 7% | 68.23 |  |
| Nup60_hc | 7% | 36.48 |  |
| Nup1m_hc | 9% | 79.15 |  |
| Nup2_hc | 12% | 62.76 |  |
| Nsp1m_hc | 4% | 67.81 |  |
| Nup145ns | 9% | 38.95 |  |
| Nup100s | 6% | 42.75 |  |
| Nup116s | 0% | 42.22 |  |

For the Phos_FPD scenario we identified 3168 phosphorylation sites inside the NPC (note that Nup49 is not listed in the FPD database). A hypothetical simultaneous phosphorylation of all these residues results in an NPC with a significant negative charge (net charge = -5824e) (see table s7). Both the net charge and hydrophobicity values for Phos_FPD are in between the corresponding values for the wild type and Phos_N NPCs (see table s5 and s7). Therefore, we can predict the density distribution for the Phos_FPD NPC to be intermediate between the Phos_N and wild type from Figure 4. Since the passive transport depends on the FG-Nup density inside the NPC, we can state that the Phos_FPD NPC will inhibit the translocation of 8.5 nm inert particle as a less dense Phos_N NPC does not allow translocation of these inert particles (Figure 7b) in the first place. In addition, the transport rate of the 4 nm inert particle for Phos_FPD is expected to be in between the wildtype and Phos_N NPCs (Figure s1).

Unlike passive transport, the prediction for active transport is not so straight-forward because of the electrostatic and hydrophobic interactions in addition to the steric interaction between the Kaps and the NPC. Therefore, we performed MD simulations for Kap transport through a Phos_FPD NPC. This simulation revealed that none of the Kaps translocated through the Phos_FPD NPC (see Figure s2). Thus, to summarize, the Phos_N NPC transport selectivity should resemble that of the Phos_FPD NPC depicted in Figure 8.

**Table s7.** Physical properties for the Phos_FPD NPC.

| **Forcefield** | **+ve charge** | **-ve charge** | **Net Charge** | **Net hydrophobicity** | **Phosphorylated AA** |
| --- | --- | --- | --- | --- | --- |
| Phos_FPD | 8072 | -13896 | -5824 | 42108.34 | 3168 |


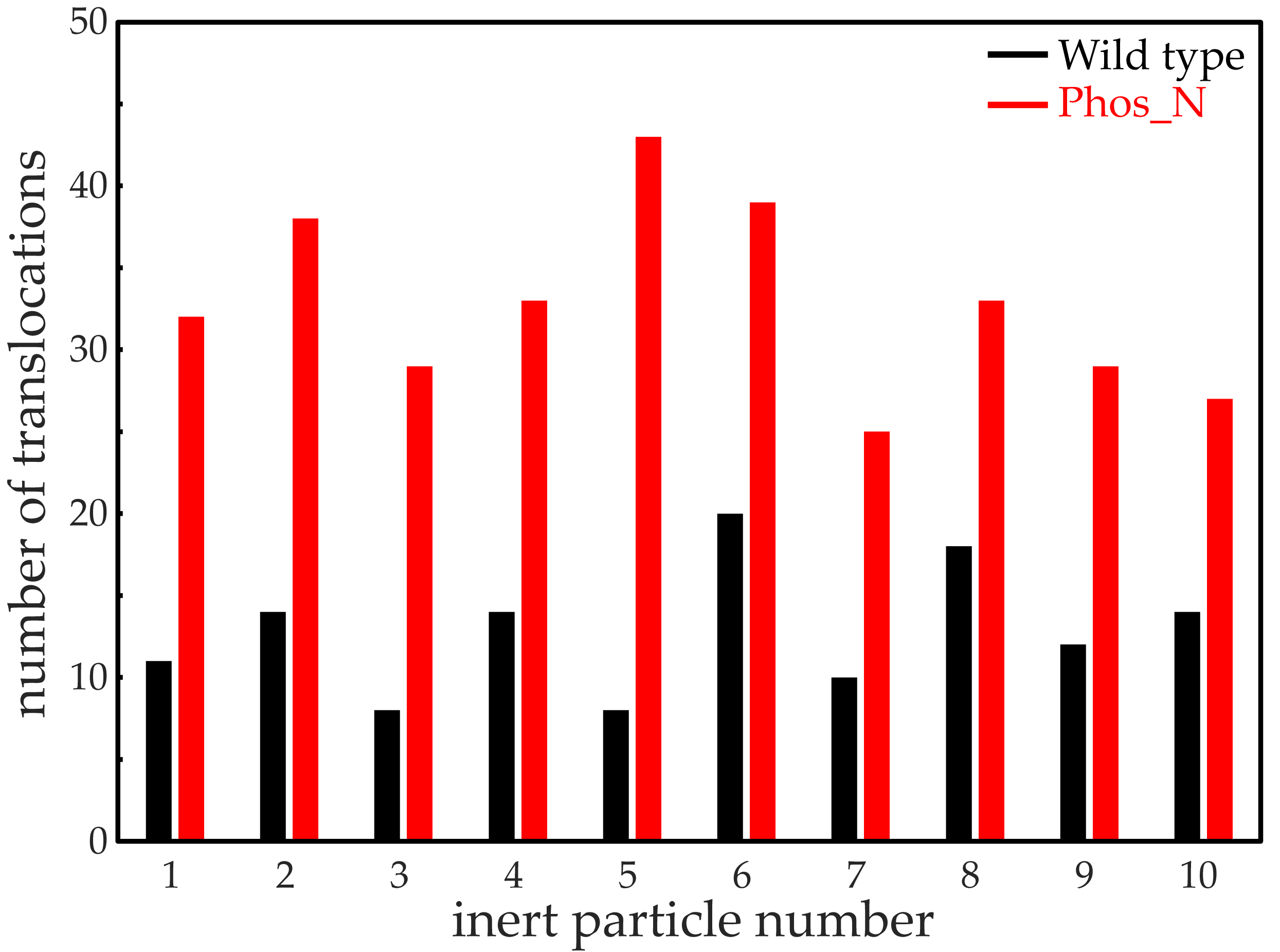


**Figure s1.** The number of translocations of each inert particle (within 2 μs of simulation time) in a wild type (black) and Phos_N (red) NPC. The number of translocations in Phos_N is on average ~2.5 times higher than that in a wild type NPC, indicating increased kinetics for passive transport in a phosphorylated NPC.


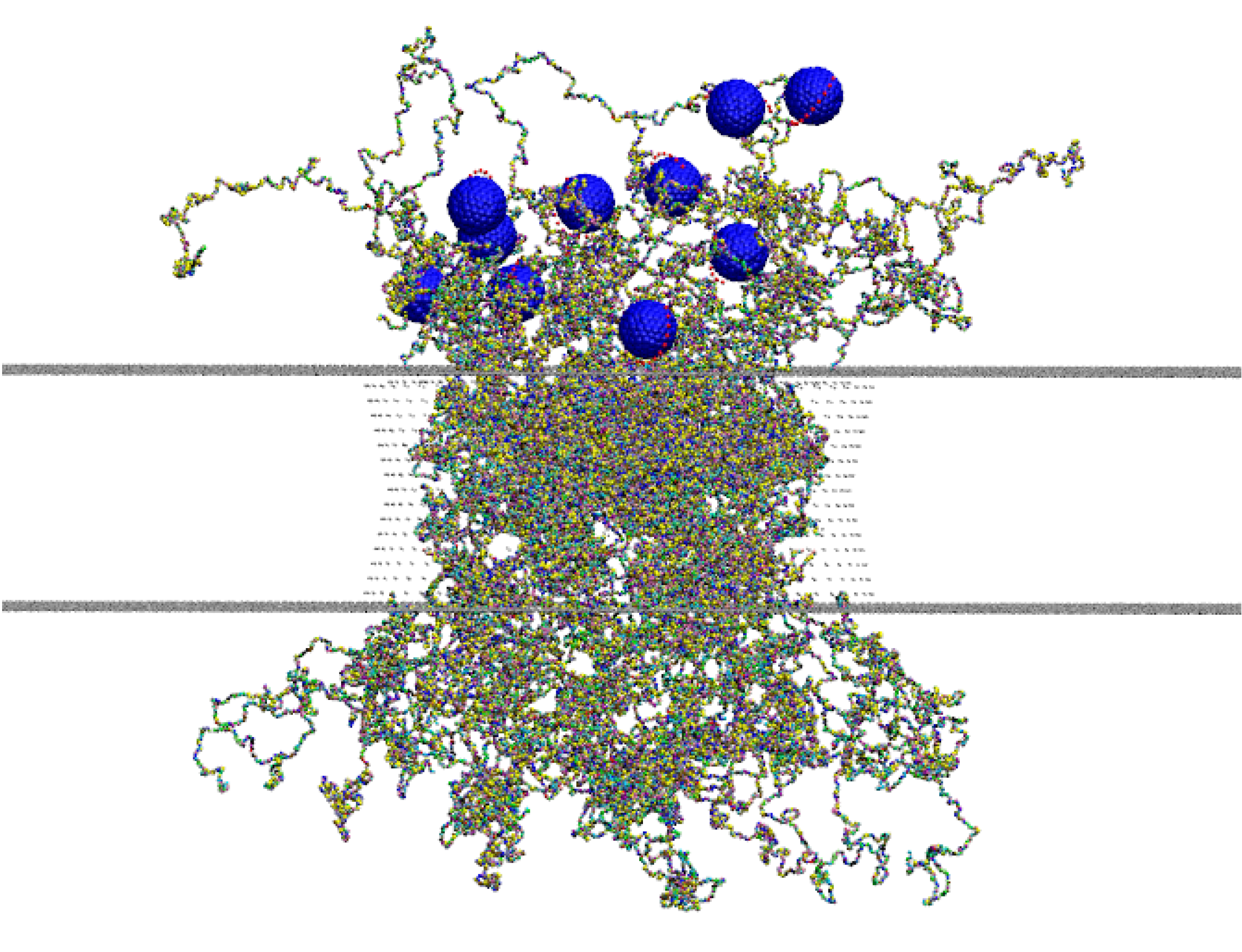


**Figure s2.** Snapshots at *t* = 2 μs of the FG-Nups and model Kap95 particles inside a Phos_FPD NPC. The Kap95 particles are shown in blue with the red hydrophobic binding spots on its surface. The 20 different amino acids of the FG-Nups are represented by different colors. The size of the scaffold beads (grey) is scaled down to make the Kap particles better visible.
